# A Bayesian decision fusion approach for microRNA target prediction

**DOI:** 10.1186/1471-2164-13-S8-S13

**Published:** 2012-12-17

**Authors:** Dong Yue, Maozu Guo, Yidong Chen, Yufei Huang

**Affiliations:** 1 Department of Electrical and Computer Engineering, University of Texas at San Antonio, San Antonio, Texas 78249, USA; 2 Department of Computer Science and Engineering, Harbin Institute of Technology, Harbin 150001, China; 3 Department of Epidemiology and Biostatistics, University of Texas Health Science Center at San Antonio, San Antonio, Texas 78229, USA

## Abstract

MicroRNAs (miRNAs) are 19-25 nucleotides non-coding RNAs known to have important post-transcriptional regulatory functions. The computational target prediction algorithm is vital to effective experimental testing. However, since different existing algorithms rely on different features and classifiers, there is a poor agreement among the results of different algorithms. To benefit from the advantages of different algorithms, we proposed an algorithm called BCmicrO that combines the prediction of different algorithms with Bayesian Network. BCmicrO was evaluated using the training data and the proteomic data. The results show that BCmicrO improves both the sensitivity and the specificity of each individual algorithm. All the related materials including genome-wide prediction of human targets and a web-based tool are available at http://compgenomics.utsa.edu/gene/gene_1.php.

## Background

Gene regulation in human genome assumes multiple modes including transcriptional regulation by the regulatory proteins or transcription factors (TFs), and post-transcriptional regulation by including most notably microRNA (miRNA). MiRNA is a small non-coding RNA that has been discovered to repress transcription and/or protein translation of hundreds of genes by binding to the 3' Untranslated Region (UTR) of target genes [1,2]. Understanding the functions and regulatory mechanisms of miRNA comprises one of the most active areas of research; such understanding will greatly advance our knowledge about the complexity of gene regulation and will help us to identify new therapeutic targets for effective treatment of various diseases.

Identifying miRNAs' target genes is an important first step in elucidating its function. Past work produced many target prediction algorithms based on miRNA-target sequence paring including TargetScan [3-5], miRanda [6,7], PicTar [8], mirTarget [9,10], PITA [11], DianamicroT [12] and others [13-21]. However, the prediction results of existing algorithms are still of low precision (i.e., low percentage of true targets among the predicted targets) and poor sensitivity (i.e., small percentage of true targets being predicted). In a recent study [22], Bartel et al., validated the prediction results of TargetScan, miRanda, PicTar, and PITA using a mass spectrometry (MS) approach. It was found that two thirds of their predicted targets appeared to be false positives, indicating a precision of only about 30%. As a result, the existing algorithms still cannot be used as target screening for subsequent bench testing.

There seems to be a poor agreement between the results of different algorithms and yet they achieve similar performance; this fact indicates that different algorithms rely on different mechanisms in making prediction, each of which has its own advantages. Indeed, the aforementioned sequence-based algorithms make predictions based on various important features of miRNA and mRNA nucleotide sequence interaction. Although a few important features including "seed region complementary", "binding free energy", and "sequence conservation" are among the most common adopted ones, different algorithms do utilize different sets of features. The differences in features and classifiers contribute to the differences in their prediction results. It is therefore desirable to integrate the predictions of different algorithms in order to combine their different advantages.

To do so, we propose a Bayesian decision fusion algorithm, BCmicrO. The goal of this algorithm is to improve the performance of existing target prediction algorithms. BCmicrO explicitly models the distributions of prediction results for each algorithm based on a training dataset composed of carefully constructed positive and negative miRNA-target pairs. These distributions capture the distinctions among different algorithms and weigh the differences at the decision level. With these distributions, the integration of different decisions is carried out based on Bayesian Network (BN). We tested the performance BCmicrO (combining TargetScan, miRanda, PicTar, mirTarget, PITA, and DianamicroT) with our training data, and validate it on the proteomics data. BCmicrO show clear improvement.

## Methods

### Overview of BCmicrO

The goal of BCmicrO is to generate the probability of an mRNA to be the target of a mRNA by integrating the predictions of different existing algorithms. In this paper, we focus on integrating TargetScan, miRanda, PicTar, mirTarget, PITA and Diana-microT's prediction scores. It should be noted that predictions from additional algorithms can be included in a similar fashion. TargetScan utilizes mainly seed region complementary and sequence conservation features for identifying potential binding sites and also applies a linear regression model to combine UTR features including 3' pairing score, local AU content, and distance from nearest 3'UTR terminus to produce a prediction context score for a UTR. On the other hand, miRanda relies on nucleotide complementariness and binding free energy in making the prediction. In contract, PicTar assumes a Hidden Markov Model (HMM) for seed region complementary and binding free energy to predict the potential binding sites. MirTarget is a SVM based algorithm with 113 features defined for a miRNA and target pairs. The key of PITA is a novel miRNA-target interaction model, based on the experimental observation - a strong secondary structure formed by 3'UTR itself will prevent the binding of miRNA. Diana-microT is a rule based miRNA target prediction algorithm applying a modified dynamic programming algorithm to determine the minimum free energy for each segment with a miRNA.

**Figure 1:**
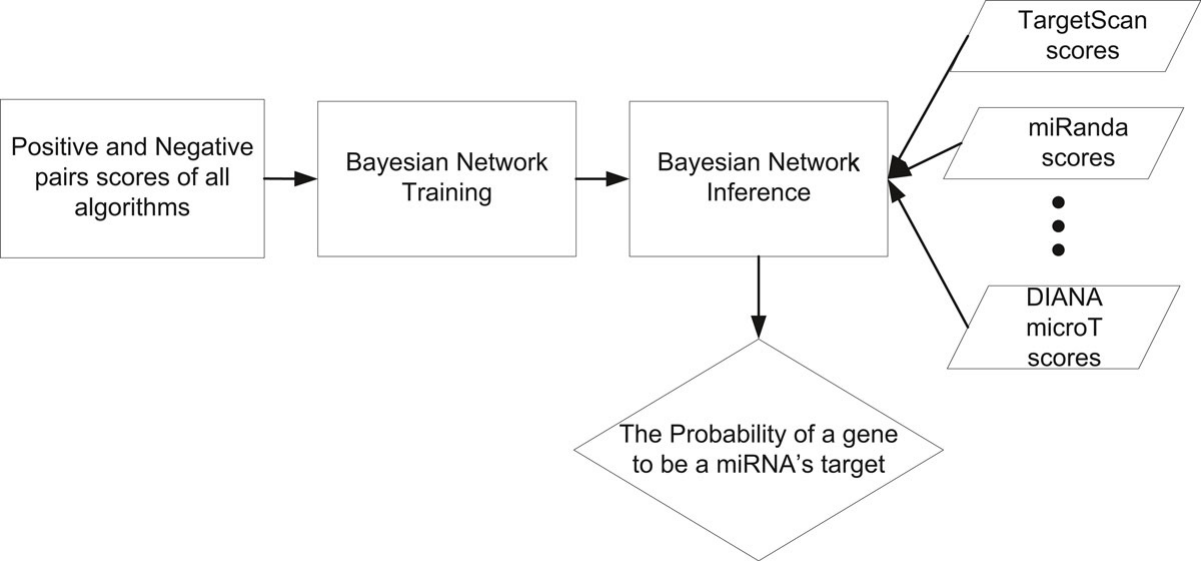
**The flow chart of BCmicrO**. During training, the distributions of the positive and negative miRNA-target pairs are acquired from the training data and are combined with the Bayesian Network model. To generate the probability of a potential target, TargetScan, miRanda, PicTar, mirTarget, PITA and Diana-microT scores have to be obtained.

The flow chart of BCmicrO is shown in Figure 1, which includes training and prediction. During training, the distributions of the positive and negative miRNA-target pairs are fitted from the training data and the Bayesian Network model is inferred. For prediction, the TargetScan, miRanda, PicTar, mirTarget, PITA and Diana-microT scores of a tested mRNA are first acquired and then feed into the trained BCmicrO to generate the probability of being target.

### Model formulation

Genome-wide predictions of TargetScan, miRanda, PicTar, mirTarget, PITA and Diana-microT are all reported in terms of scores. Particularly, TargetScan predicts miRNA's potential binding sites in the mRNA's 3' UTR, a context score is calculated for each site and the total context score is computed to represent the confidence of an mRNA to be a target. MiRanda indentifies all possible target sites for an mRNA and the highest target site score is selected to represent the confidence of the corresponding mRNA being a target. PicTar and other algorithms also compute a score reflecting the likelihood that the mRNA is a target.

To integrate these scores, BCmicrO adopts a BN model. BN is also known as directed graphical models, where the links of the graphs have a particular directionality indicated by arrows. The unique feature of BN is that the joint distribution over all of the random variables can be decomposed into a product of factors, each depending only on a subset of the variables [23].

**Figure 2:**
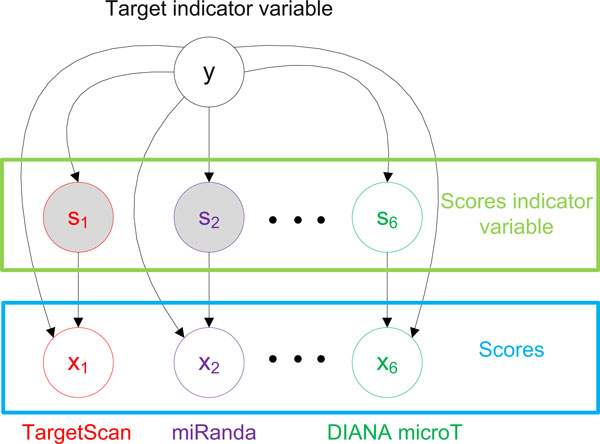
**Graphical model of BCmicrO**. *y *is an indicator, and *y *= 1 when the mRNA is the miRNA target. *x*_1_,*x*_2_,...,*x*_6 _are TargetScan, miRanda, PicTar, mirTarget, PITA and Diana-microT scores, respectively. *s*_1_,*s*_2_,...,*s*_3 _are indicator variables to show whether TargetScan, miRanda, PicTar, mirTarget, PITA and Diana-microT have scores.

The structure of the BN model is shown in Figure 2. Specifically, let *x*_1_,*x*_2_,...*x*_6 _denote the scores of a miRNA-mRNA pair by TargetScan, miRanda, PicTar, mirTarget, PITA and Diana-microT, respectively. Also, set *y *as an indicator variable such that *y *= 1, if the mRNA is a real miRNA target, and *y *= 0, otherwise. The goal of BCmicrO is to calculate *P *(*y *= 1|*x*_1_, *x*_2_, ..., *x*_6_), the posterior probability of the mRNA to be the miRNA's target given the TargetScan, miRanda, PicTar, mirTarget, PITA and Diana-microT scores. In reality, not all six scores are available for a miRNA-mRNA pair. Commonly, each algorithm only provides the prediction scores meeting a cutoff threshold. Therefore, we introduce the score indicators *s*_1_, *s*_2_,...,*s*_6 _to denote whether TargetScan, miRanda, PicTar mirTarget, PITA and Diana-microT report scores, or *s_i _*= 1(*i *∈ {1, 2, ..., 6}) if the algorithm i reports a score, and *s_i _*= 0 otherwise. Also note that *x_i _*may be a score or no score (NaN) because of the cutoff value that mentioned before. The posterior probability can be expressed based on the BN model as

(1) $$p\left(y=1|x_{1},x_{2},...,x_{6}\right)=\frac{p\left(x_{1},x_{2},...,x_{6}|y=1\right)p\left(y=1\right)}{p\left(x_{1},x_{2},...,x_{6}|y=1\right)p\left(y=1\right)+p\left(x_{1},x_{2},...,x_{6}|y=0\right)p\left(y=0\right)}$$

where *p*(*y *= 1) is the prior probability of an mRNA being a target and *p*(*x*_1_, *x*_2_, ...,*x*_6_|*y*) is the likelihood function. It is noted from the graphical model that given y, *x*_1_,*x*_2_,...,*x*_6 _are conditional independent and thus

(2) $$p\left(x_{1},x_{2},\;...,x_{6}|y\right)=p\left(x_{1}|y\right)p\left(x_{2}|y\right)...p\left(x_{6}|y\right)$$

where

(3) $$p\left(x_{i}|y\right)=\sum_{s_{i}\in \left\{1,0\right\}}p\left(x_{i},s_{i}|y\right)=\sum_{s_{i}\in \left\{1,0\right\}}p\left(x_{i}|s_{i},y\right)p\left(s_{i}|y\right)$$

It becomes clear that *p*(*y *= 1|*x*_1_, *x*_2_, ..., *x*_6_) can be calculated from (1)-(3) once we have the conditional distributions *p*(*x_i_*|*s_i_, y*) and *p*(*s_i_*|*y*), for *s_i _*∈ {0, 1}, *y *∈ {0, 1}, and *i *∈ {1, 2, ...,6}. In addition, based on Graphical model (Figure 2), given y, the conditional distributions of different algorithms are independent, such as *p*(*x*_1_|*s*_1_, *y*) is conditional independent of *p*(*x*_2_|*s*_2_, *y*). In the later section, we will discuss the process of acquiring all the above conditional probabilities in detail.

### Training data construction

Since the desired conditional distributions depend on y, i.e. the true target status of the mRNA, we need to collect high confidence positive and negative miRNA-target pairs as training data.

**The positive miRNA-target pairs **are collected from miRecords, which stores high-quality experimentally verified miRNA targets [24]. Only mammalian - human, mouse and rat records (852 records) are of our interest, since our goal is to identify mammalian miRNA target. Moreover, Karginov et al. [25] found the mRNAs whose association with Argonaute 2 (Ago2) increased upon miRNA over-expression were more likely targeted by miRNA, and 293 mRNAs were obtained as miR-124's targets. Lastly, 22 experimentally validated miR-124 targets are also collected from paper [25]. We combined the above positive miRNA-target pairs, removed the duplicate records, and ended up with a set of 929 positive miRNA-target pairs.

**The negative miRNA-target pairs **are currently unavailable in any annotated database. We constructed our negative database from two sources. First, it is known that negative targets are mostly up-regulated under miRNA over-expression. Therefore, first of all, negative targets were extracted as the up-regulated genes in 20 microarray data due to miRNA over-expression from NCBI Gene Expression Omnibus (GEO). To assure the high quality of negative data, we only chose the most confident up-regulated genes by restricting the differential expression p value, the fold change and consistency of the samples over time whenever available. To be more specific, the differential expression p value of the negative target must be less than 0.001 to ensure it is differentially expressed and the fold change (FC) of the negative target must be greater than 1.5 to ensure it is not down-regulated. In this process, 3542 negative miRNA-target pairs were gained. This is a high confident negative set compared to those miRNA-gene pairs with un-changed expression or random sampling.

Second, we focus on the existing results of miR-124 using immunoprecipitation (IP) of Ago2, since this technology has both higher sensitivity and specificity than other technologies including microarray and proteomics. Therefore, we obtained 19780 negative miR-124 targets by excluding 22 luciferase validated targets validated and 293 miR-124 target genes predicted in [25]. In the end, 23319 negative miRNA-target pairs were acquired.

The prediction scores of the positive and negative pairs for the three algorithms were subsequently obtained. The TargetScan (v5.1) scores were downloaded from web site (http://www.TargetScan.org/) [4,5]. miRanda (2008 Sept) scores were downloaded from web site (http://www.microrna.org) [6,7]; PicTar (2006) target predictions were downloaded from web site (http://PicTar.mdc-berlin.de/) [8]; mirTarget prediction results were downloaded from website (http://mirdb.org/miRDB/download.html) [9,10]; PITA scores were downloaded from (http://genie.weizmann.ac.il/pubs/mir07/index.html) [11]. Diana-microT scores are downloaded from (http://diana.cslab.ece.ntua.gr/microT/) [12].

### Training of the conditional distributions

***1***. *p*(*x_i _*= *score*|*s_i _*= 1, *y *= 1), *i *∈ {1, 2, ...6}

**Figure 3:**
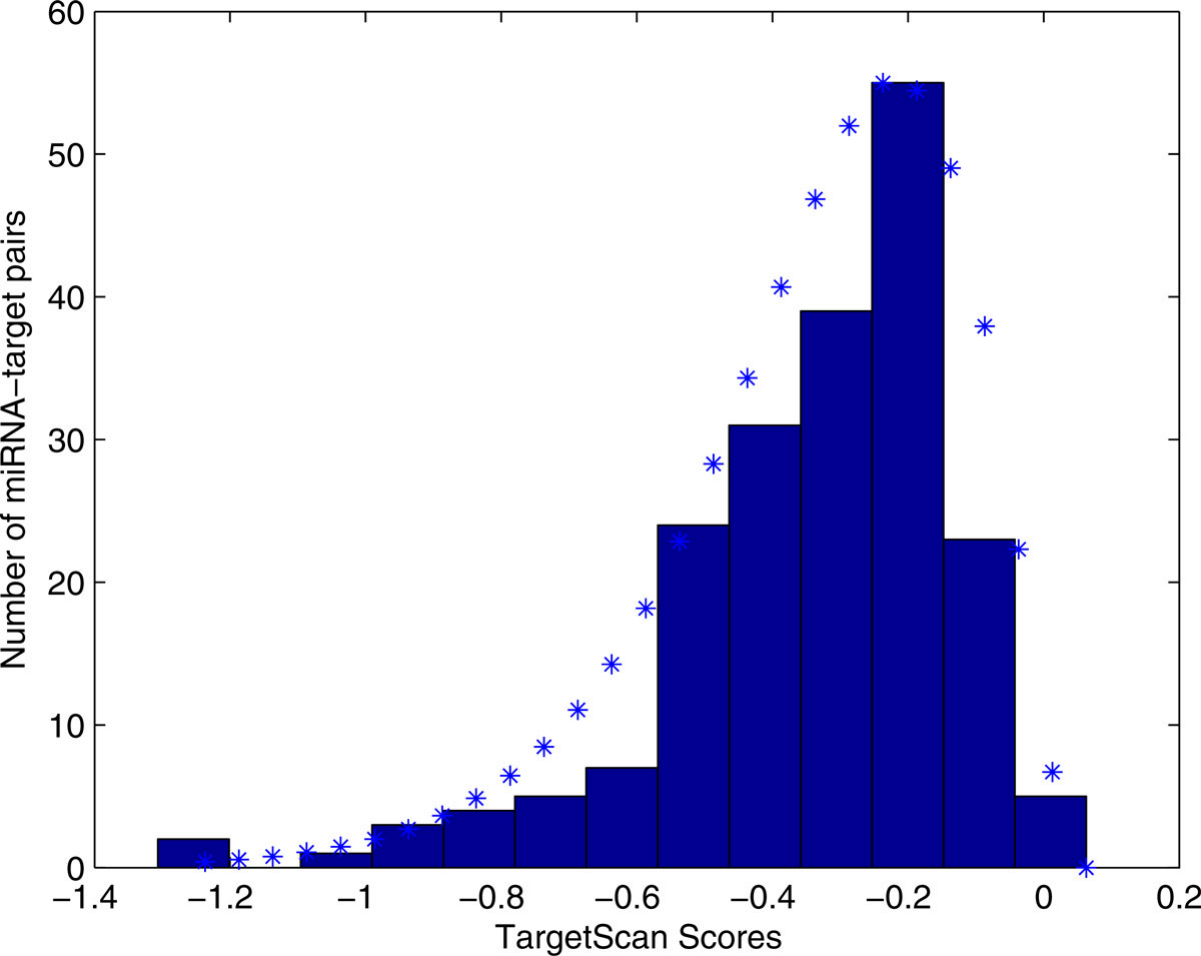
**The histogram of the positive pairs' TargetScan scores and the fitted distribution**. In the training data, 199 TargetScan scores for the positive miRNA-target pairs are obtained. Its histogram is fitted with Gamma distribution. The fitted distribution is represent by blue stars.

The meaning of *p*(*x_i _*= *score*|*s_i _*= 1, *y *= 1) is the probability of a miRNA-target pair's score of algorithm *i *given this pair is a positive pair and has a score. Note that "*x_i _*= *score*" means *x_i _*has score. To find this conditional distribution, we should obtain the positive miRNA-target pairs with scores. To this end, we searched the positive miRNA-target pairs in each algorithm prediction results. Specifically, for TargetScan prediction results, 199 scores were obtained for the positive miRNA-target pairs and the histogram is shown in Figure 3. Upon flipping the histogram horizontally at its maximum score, a Gamma distribution was fitted. Maximum likelihood estimator (MLE) was used to estimate the parameters of the Gamma distribution. *p*(*x_i _*= *score*|*s_i _*= 1, *y *= 1) (*i *= 2 to 6), can be determined similarly, with a total of 278,175, 214, 631 and 396 positive pairs with scores for miRanda (Additional file 1), PicTar (Additional file 2), mirTarget (Additional file 3), PITA (Additional file 4), and Diana-microT (Additional file 5), respectively.

***2***. *p*(*x_i _*= *score*|*s_i _*= 1, *y *= 0), *i *∈ {1, 2, ... 6}

**Figure 4:**
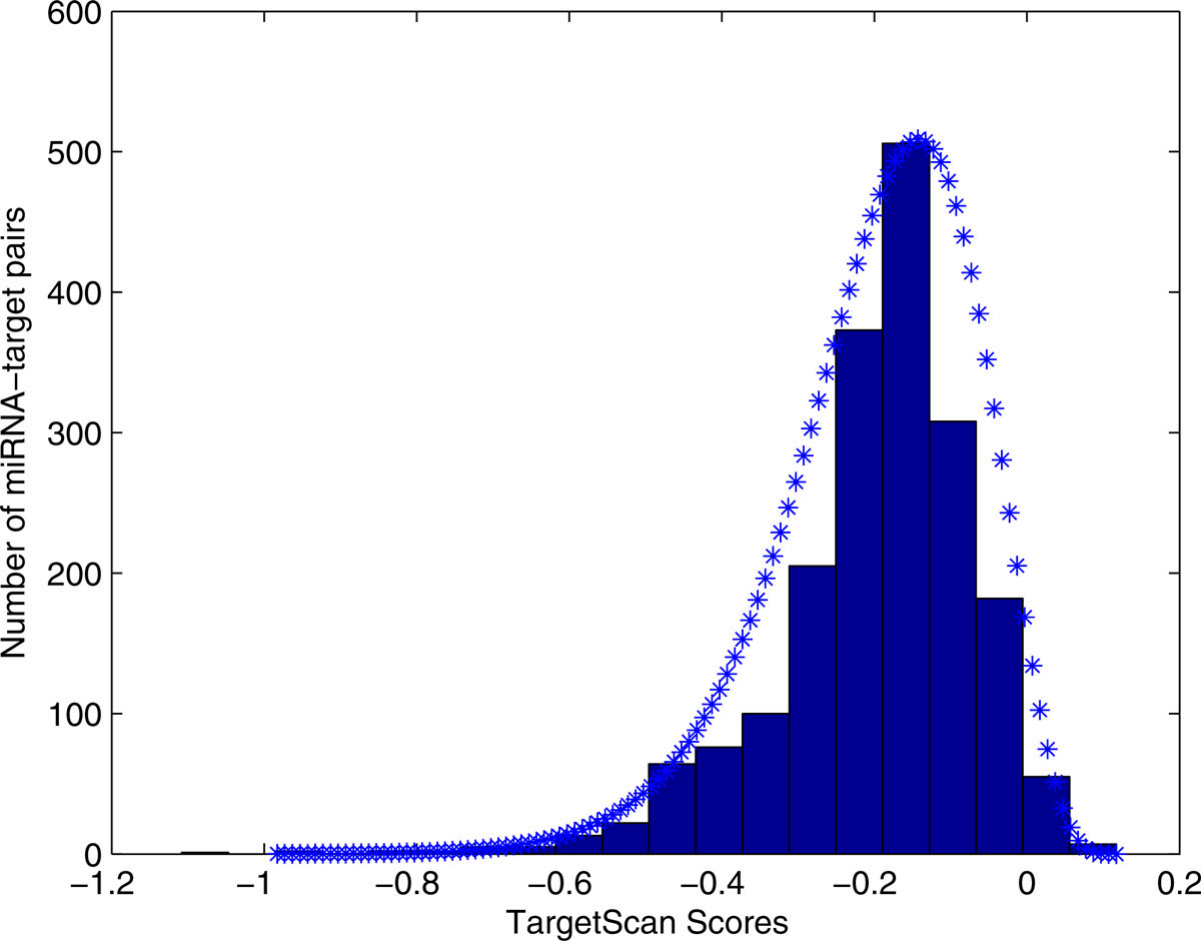
**The histogram of the negative pairs' TargetScan scores and the fitted distribution**. In the training data, 1928 TargetScan scores for the negative miRNA-target pairs are obtained. Its histogram is fitted with Gamma distribution. The fitted distribution is represent by blue stars.

*p*(*x_i _*= *score*|*s_i _*= 1, *y *= 0) is the probability of a miRNA-target pair's score *x_i _*from algorithm *i*, given this pair is a negative pair and has a reported score. Similar as *p*(*x_i _*= *score*|*s_i _*= 1, *y *= 1), we searched the negative miRNA-target pairs prediction results. 1928 negative miRNA-target pairs with TargetScan scores were acquired. The Gamma distribution was fitted to the scores (Figure 4). For miRanda (Additional file 6), PicTar (Additional file 7), mirTarget (Additional file 8), PITA (Additional file 9), and Diana-microT (Additional file 10), 1230, 613,436, 8831 and 3254 scores for the negative pairs were obtained, respectively.

***3***. *p*(*s_i_*|*y*), *i *∈ {1, 2, ... 6}, *s_i _*∈ {0, 1}, *y *∈ {0, 1}

*p*(*s_i _*= 0|*y *= 0), *p*(*s_i _*= 0|*y *= 1), *p*(*s_i _*= 1|*y *= 0), and *p*(*s_i _*= 1|*y *= 1) are the true negative rate (TNR), false negative rate (FNR), false positive rate (FPR), and true positive rate (TPR) for each algorithm. Since the prediction is carried out genome-wide, they should be assessed for a data set of a similar composition of positive and negative targets for human genome. The real miR-124 targets are retrieved from [25] including 22 targets validated by luciferase and 256 Net IP enrichment identified target genes, and the rest of genes are considered as the negative miR-124 targets (19780). Table 1 enlisted the estimated probabilities of each algorithm. We assume that the performance of each algorithm is consistent for all miRNAs. As a byproduct, the prior *p*(*y *= 1) is estimated from the Net IP data of miR-124 as 0.0133.

**Table 1:** TP, FP, TN and FN rate of the 3 algorithms

	TP rate	FP rate	TN rate	FN rate
TargetScan	0.4082	0.0877	0.9123	0.5918

miRanda	0.3371	0.0783	0.9217	0.6629

PicTar	0.1798	0.0390	0.9610	0.8202

mirTarget	0.2285	0.0302	0.9698	0.7715

PITA	0.7603	0.3942	0.6058	0.2397

Diana-microT	0.4045	0.1474	0.8526	0.5955

The true positive (TP), false positive (FP), true negative (TN) and false negative (FN) rate of the algorithms that used in BCmicrO.

*4. Other Conditional distributions*

It is apparent that *p*(*x_i _*= *score*|*s_i _*= 0, *y*) = 0 for all *i*. Similarly, we have *p*(*x_i _*= *NaN *| *s_i _*= 0, *y*) = 1,*p*(*x_i _*= *NaN *| *s_i _*= 1, *y*) = 0

## Result

### Test of BCmicrO on training data

**Figure 5:**
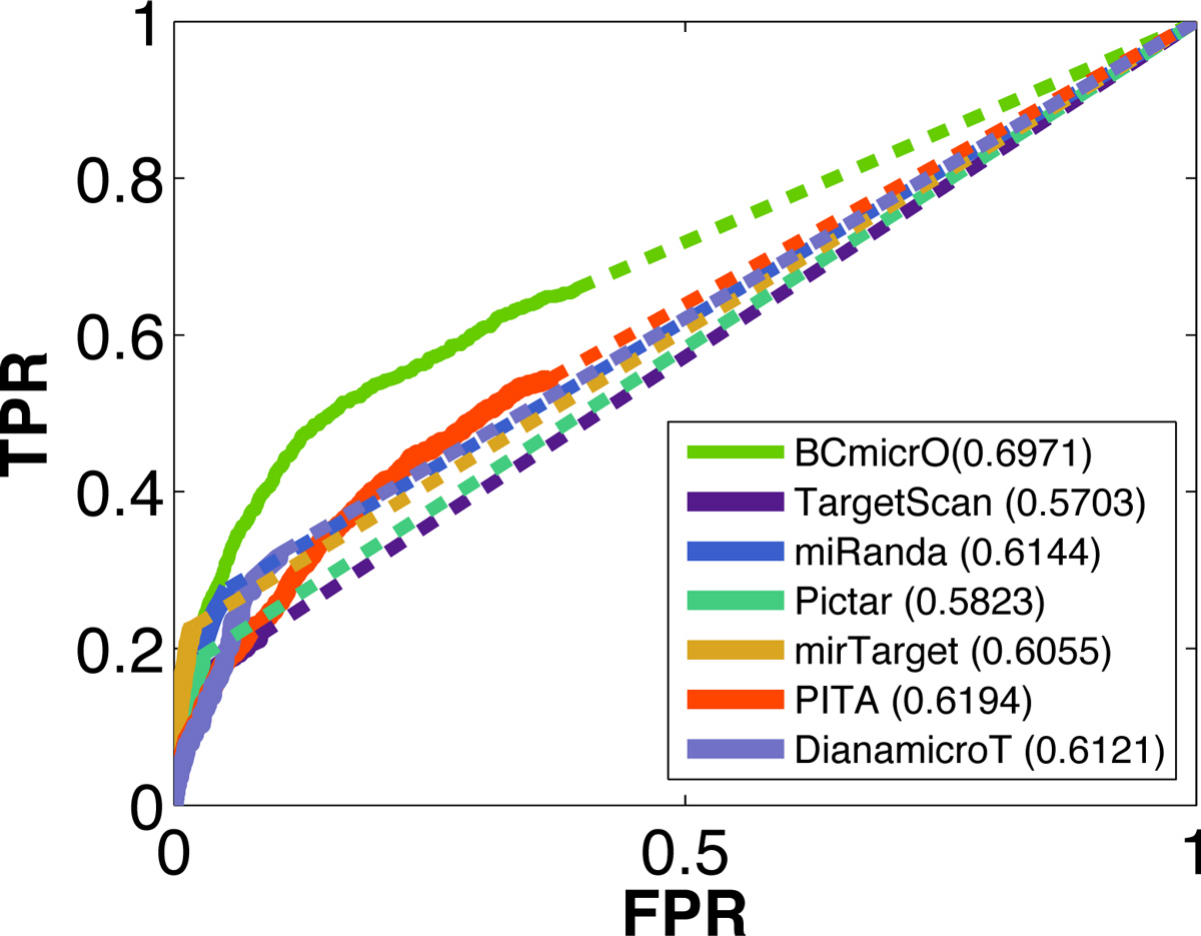
**The ROC curves of BCmicrO and other algorithms**. In order to show the performance of different methods in the training data, the ROC curves are drawn. A dash lined is applied when not all scores are available. BCmicrO achieved the largest Area Under the Curve(AUC).

**Figure 6:**
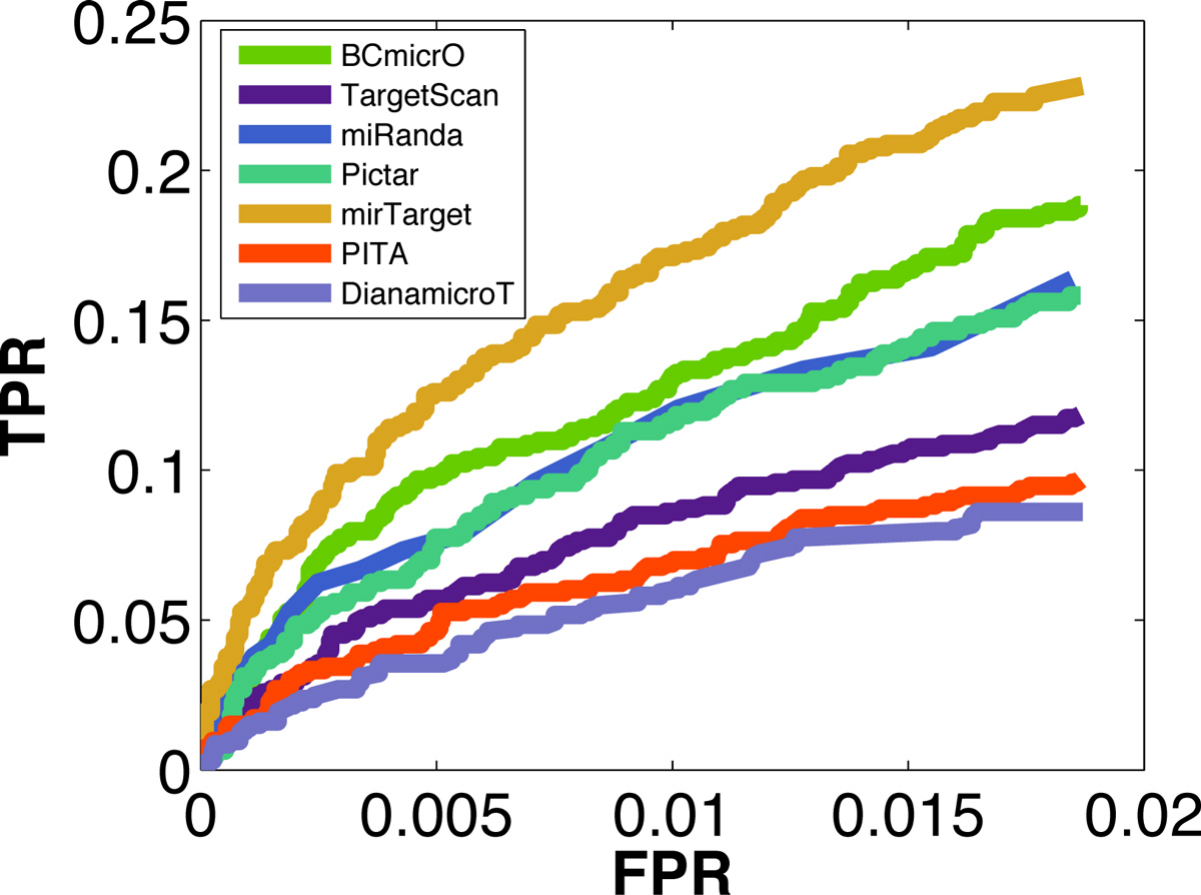
**The ROC curves in low FPR region**. The lower FPR region in Figure 5. It stops at 0.0187 - the farthest FPR that mirTarget can reach. BCmicrO is the second best in the low FPR region.

To evaluate the performance of BCmicrO, 5-fold cross validation is performed in our positive and negative training data. Each time, we trained the Bayesian Network with 4-fold training data, and predict the BCmicrO scores for the rest one fold testing data. To compare the performance of different methods, we drew the ROC curve for each algorithm as shown in Figure 5, where the Area Under the Curve (AUC) of each algorithm is also showed. Since scores are not available for all training data, a dash line is applied to interpolate the curve (Or the estimation of the algorithm's performance). As can be seen, BCmicrO has the best performance with the largest AUC (0.6971). Note also that BCmicrO has higher TPR then other algorithms when FPR is 0.05. Clearly, BCmicrO has the best sensitivity since it has the highest starting TP for the dash line. Since the ROCs are too close to each other in the lower FPR region, then we plot the ROC curve again (Figure 6) and let it stop at 0.0187 which the largest FPR that mirTarget can reach. As this figure shows, BCmicrO is the second best in the low FPR region.

### Test of BCmicrO on proteomics data

**Figure 7:**
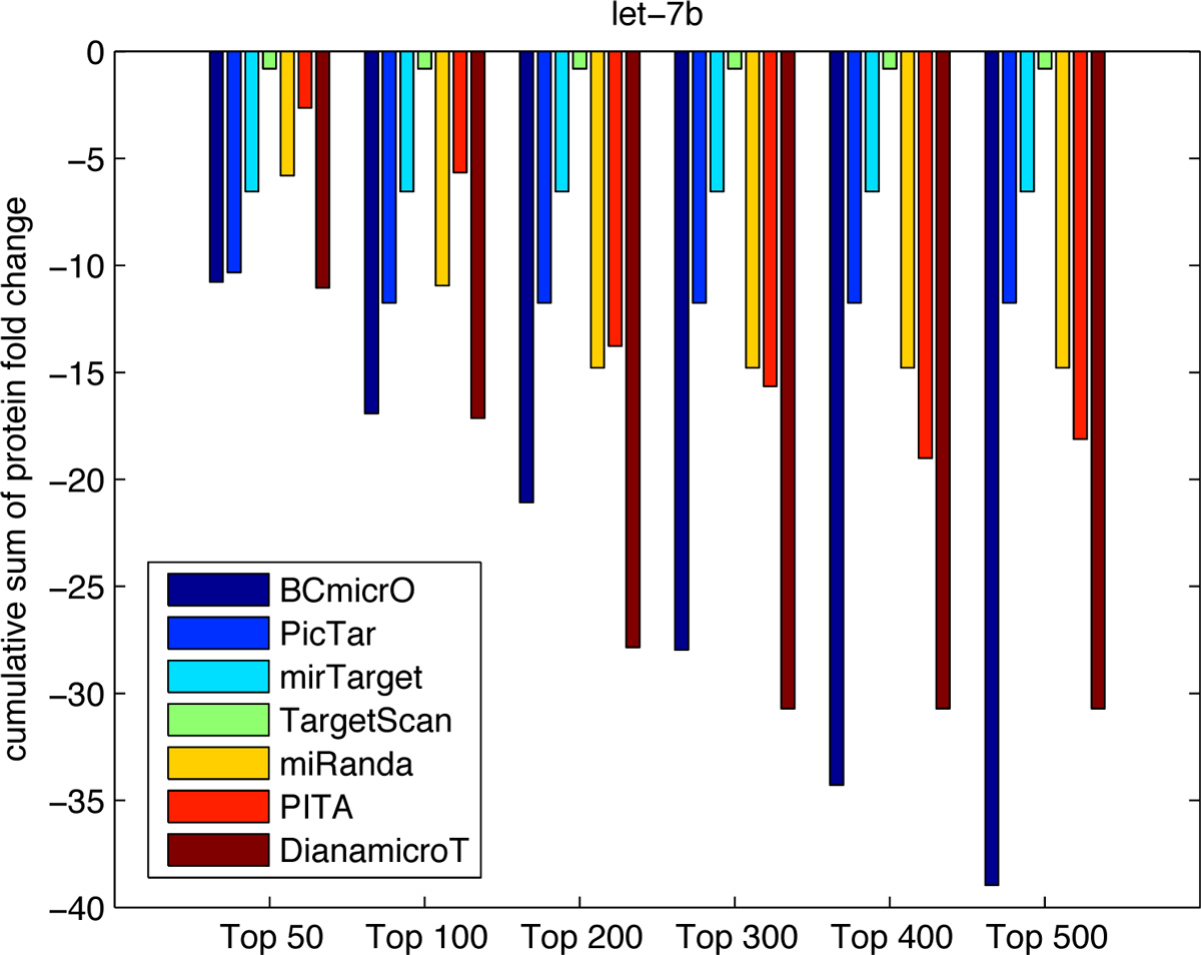
**Cumulative sum of protein fold change for different number of top ranked predictions of let-7b**. The cumulative sum of fold change and ranked predictions are shown for each algorithm for miRNA let-7b.

**Figure 8:**
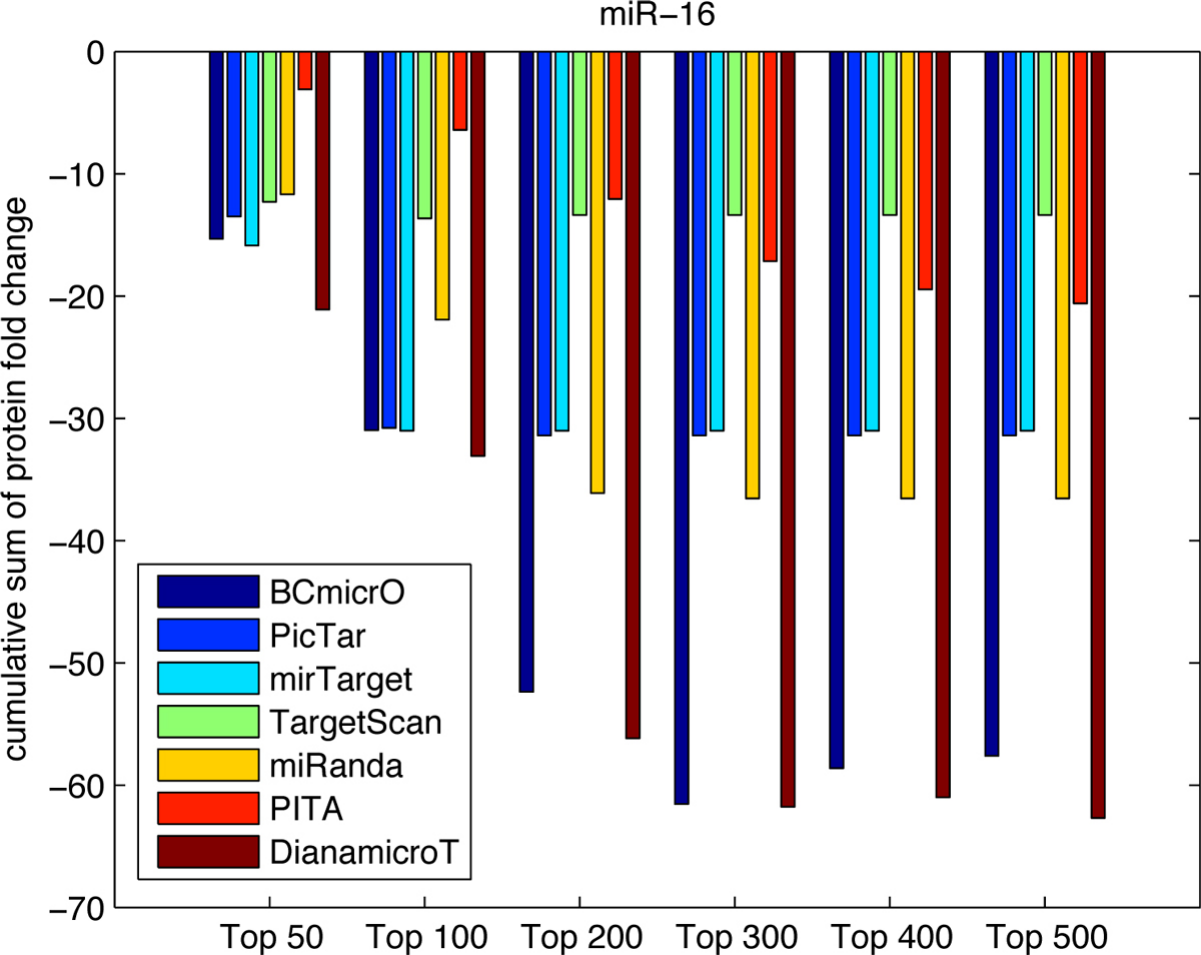
**Cumulative sum of protein fold change for different number of top ranked predictions of miR-16**. The cumulative sum of fold change and ranked predictions are shown for each algorithm for miRNA miR-16.

**Figure 9:**
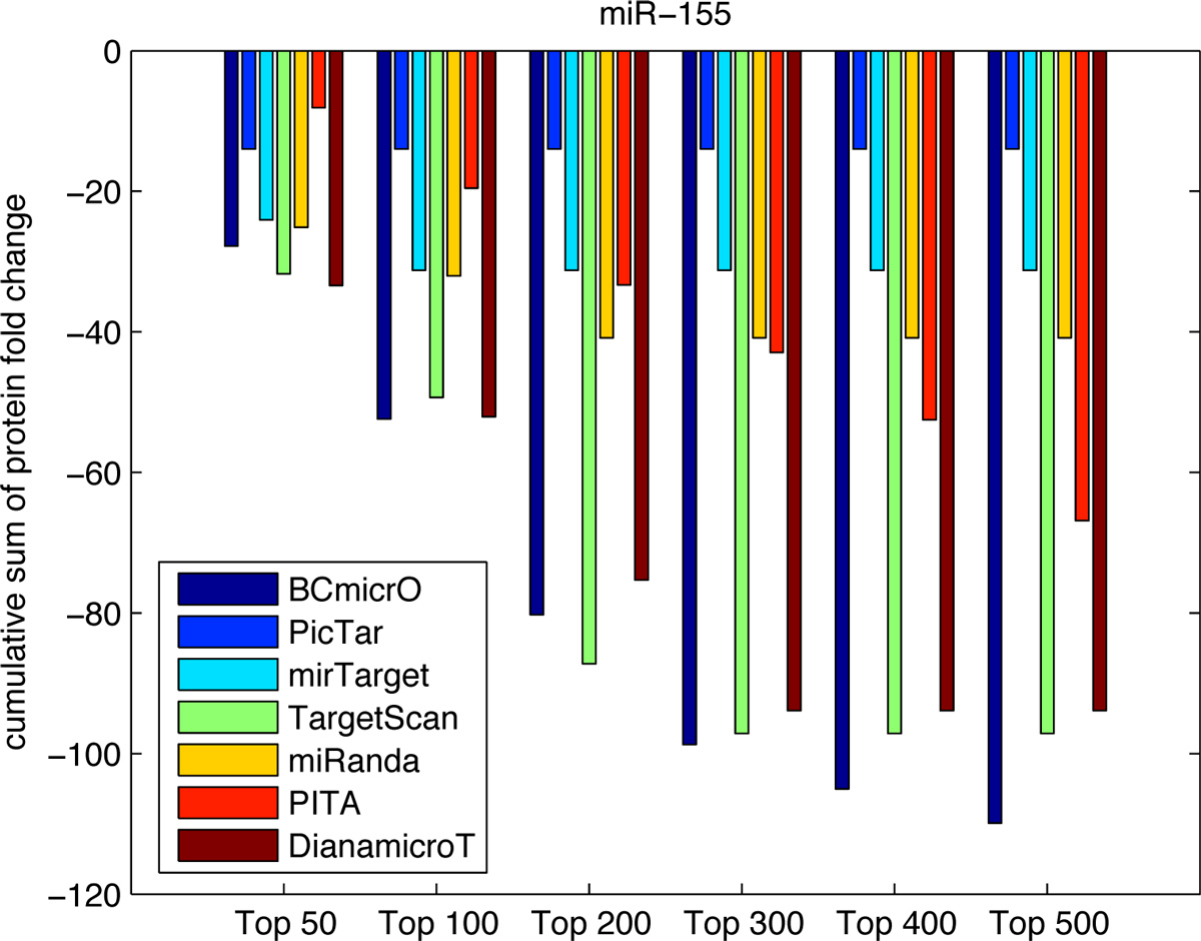
**Cumulative sum of protein fold change for different number of top ranked predictions of miR-155**. The cumulative sum of fold change and ranked predictions are shown for each algorithm for miRNA miR-155.

**Figure 10:**
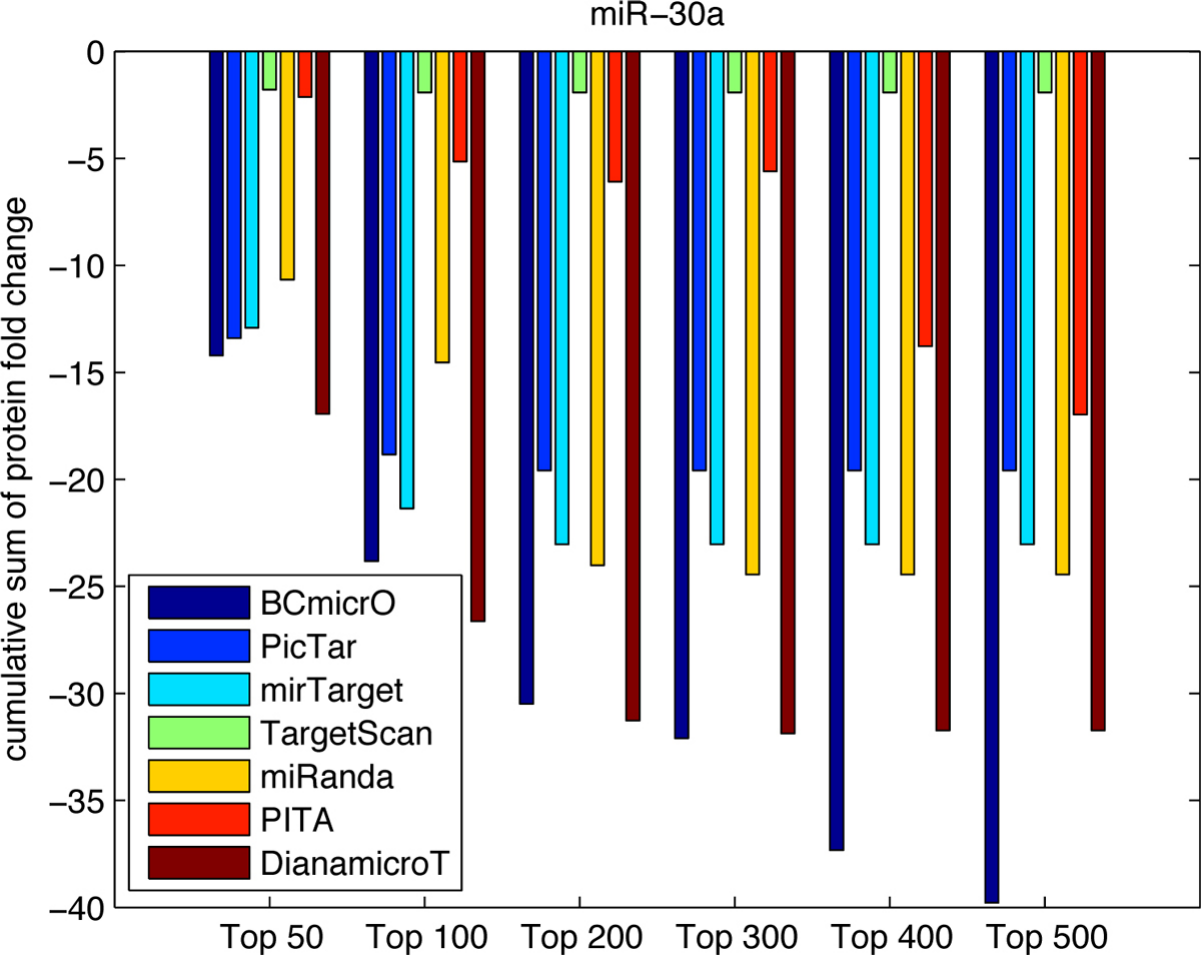
**Cumulative sum of protein fold change for different number of top ranked predictions of miR-30a**. The cumulative sum of fold change and ranked predictions are shown for each algorithm for miRNA miR-30a.

To further evaluate the performance of BCmicrO, we tested them on data not related to training data. Specifically, we consider the high throughput proteomics data, which measures the fold change of protein expression due to the over-expression of let-7b, miR-16, miR-30a and miR-155 by stable-isotope-labeling-of-amino-acids-in culture (SILAC) quantified by LC/MS [22,26]. Protein level down expression is a direct indication of miRNA regulation, since protein inhibition is regarded as primary mode of miRNA down regulation. A protein with a larger down-fold indicates that the corresponding gene is more likely to be a true target. In Figure 7, 8, 9, 10, the cumulative sum of protein fold change vs. ranked predictions are shown for each algorithm. A better algorithm is expected to show faster drops in the cumulative sum of fold change. First of all, BCmicrO has the lowest cumulative sum in the larger top ranked predictions, such as top 300, 400 and 500 in let-7b (Figure 7), miR-155 (Figure 9) and miR-30a (Figure 10). This fact is consistent with our purpose of BCmicrO - better performance than other algorithms. Overall, the performance of BCmicrO is consistent; it is always among the best performers in all cases.

We further quantified the performance of each algorithm. A better algorithm should have a cumulative sum curve with two characteristics: 1) it drops faster at the beginning, signifying a higher precision, and 2) it has the highest overall drop. Therefore, we calculated the area under the cumulative sum curve as a measurement of the performance for each algorithm

(4) $$A\left(n\right)=\overset{n}{\underset{t=1}{\sum }}c\left(t\right)$$

where *n *is the rank of the predictions and *c*(*t*) denotes the cumulative sum at rank *t*. In table 2, 3, 4, 5, we calculate *A*(*n*) for *n *∈ {100, 200, 300, 400, 500} for each algorithm. The lower *A*(*n*), the better the performance. In sum, BCmicrO has a clear advantage over the rest algorithm in top 400 and top 500 (Table 2 - Table 6). In addition, BCmcirO has the lowest cumulative sum at top 300 of miR-155 (Table 4). To quantify the consistency of the algorithm, we calculate the average cumulative sum of protein fold change in all miRNAs tests:

**Table 2:** Cumulative protein down-fold for miR-let-7b

	100	200	300	400	500
BCmicrO	-1010	-2931	-5369	-8387	-12164

PicTar	-626.6	-626.6	-626.6	-626.6	-626.6

mirTarget	-79.2	-79.2	-79.2	-79.2	-79.2

miRanda	-595.7	-1439	-1439	-1439	-1439

PITA	-289.9	-1281	-2690	-4394	-6221

Diana-microT	-1021	-3393	-5956	-5956	-5956

TargetScan	-6.9	-6.9	-6.9	-6.9	-6.9

**Table 3:** Cumulative protein down-fold for miR-16

	100	200	300	400	500
BCmicrO	-1644	-5935	-11682	-17803	-23623

PicTar	-1564	-1720	-1720	-1720	-1720

mirTarget	-1402	-1402	-1402	-1402	-1402

miRanda	-1170	-4237	-7143	-7143	-7143

PITA	-225.2	-1194	-2714	-4745	-6813

Diana-microT	-2058	-6435	-12499	-18696	-23338

TargetScan	-1062	-1102	-1102	-1102	-1102

**Table 4:** Cumulative protein down-fold for miR-155

	100	200	300	400	500
BCmicrO	-3001	-9657	-18582	-28624	-39434

PicTar	-217	-217	-217	-217	-217

mirTarget	-1061	-1061	-1061	-1061	-1061

miRanda	-2193	-4683	-4683	-4683	-4683

PITA	-868	-3627	-7210	-12089	-18034

Diana-microT	-3050	-9657	-15804	-15804	-15804

TargetScan	-3059	-10008	-15808	-15808	-15808

**Table 5:** Cumulative protein down-fold for miR-30a

	100	200	300	400	500
BCmicrO	-1298	-3816	-6982	-10523	-14403

PicTar	-1159	-1237	-1237	-1237	-1237

mirTarget	-1186	-1432	-1432	-1432	-1432

miRanda	-1006	-3014	-3183	-3183	-3183

PITA	-218	-900	-1713	-2654	-4112

Diana-microT	-1521	-4331	-7423	-9550	-9550

TargetScan	-99.8	-99.8	-99.8	-99.8	-99.8

**Table 6:** Average cumulative protein down-fold for all miRNAs

	100	200	300	400	500
BCmicrO	-869.3	-2792	-5327	-8167	-11203

PicTar	-445.9	-475.2	-475.2	-475.2	-475.2

mirTarget	-466.1	-496.9	-496.9	-496.9	-496.9

miRanda	-620.7	-1671	-2056	-2056	-2056

PITA	-200.2	-875.3	-1791	-2985	-4397

Diana-microT	-956	-2977	-5210	-6250	-6831

TargetScan	-528.5	-1402	-2127	-2127	-2127

(5) $$F\left(n\right)=\frac{1}{4}\overset{4}{\underset{i=1}{\sum }}A_{i}\left(n\right)$$

Here, *A_i_*(*n*) is the score function in (4), and *i *means the ith miRNA. F(n) is the final score at top *n *in all miRNAs tests. The scores of F(x) is shown in Table 6. BCmicrO ranks number 1 at top 300 - 500, which clearly showed that BCmicrO can consistently provide the best prediction when compared with individual algorithms in most of the cases.

## Conclusion

We proposed a new miRNA target prediction algorithm, BCmicrO, which combines the prediction result of 6 algorithms -PicTar, mirTarget, PITA, miRanda, DianamicroT, and TargetScan, using Bayesian Network.

Performance of BCmicrO was first validated based on the training data. It shows that BCmicrO has better AUC than the other 6 algorithms and also has higher sensitivity, given the same specificity. BCmicrO was also tested on proteomic data for miR-16, let-7b, miR-155, and miR-30a. BCmicrO achieved the lowest cumulative sum of protein fold change and proven to consistently deliver the best performance. BCmicrO is of low complexity and can be easily upgraded as each constituent algorithm improves itself. Additional algorithms can be also integrated into BCmicrO in a similar fashion.

## Competing interests

The authors declare that they have no competing interests.

## Authors' contributions

DY, MG, YC, and YH conceived the idea. DY and YH worked out the detailed derivations. DY implemented the algorithm and performed the prediction. DY, MG, YC, and YH wrote the paper.

## Supplementary Material

Additional file 1 **The histogram of the positive pairs' miRanda scores and the fitted distribution**. In the training data, 278 miRanda scores for the positive miRNA-target pairs are obtained. Its histogram is fitted with Negative Binomial distribution. The fitted distribution is represent by blue stars. Click here for file

Additional file 2 **The histogram of the positive pairs' PicTar scores and the fitted distribution**. In the training data, 175 PicTar scores for the positive miRNA-target pairs are obtained. Its histogram is fitted with Gamma distribution. The fitted distribution is represent by blue stars. Click here for file

Additional file 3 **The histogram of the positive pairs' mirTarget scores and the fitted distribution**. In the training data, 214 mirTarget scores for the positive miRNA-target pairs are obtained. Its histogram is fitted with Mixture Gaussian distribution. The fitted distribution is represent by blue stars. Click here for file

Additional file 4 **The histogram of the positive pairs' PITA scores and the fitted distribution**. In the training data, 631 PITA scores for the positive miRNA-target pairs are obtained. Its histogram is fitted with Gaussian distribution. The fitted distribution is represent by blue stars. Click here for file

Additional file 5 **The histogram of the positive pairs' Diana-microT scores and the fitted distribution**. In the training data, 396 Diana-microT scores for the positive miRNA-target pairs are obtained. Its histogram is fitted with Exponential distribution. The fitted distribution is represent by blue stars. Click here for file

Additional file 6 **The histogram of the negative pairs' miRanda scores and the fitted distribution**. In the training data, 1230 miRanda scores for the negative miRNA-target pairs are obtained. Its histogram is fitted with Negative Binomial distribution. The fitted distribution is represent by blue stars. Click here for file

Additional file 7 **The histogram of the negative pairs' PicTar scores and the fitted distribution**. In the training data, 613 PicTar scores for the negative miRNA-target pairs are obtained. Its histogram is fitted with Gamma distribution. The fitted distribution is represent by blue stars. Click here for file

Additional file 8 **The histogram of the negative pairs' mirTarget scores and the fitted distribution**. In the training data, 436 mirTarget scores for the negative miRNA-target pairs are obtained. Its histogram is fitted with Exponential distribution. The fitted distribution is represent by blue stars. Click here for file

Additional file 9 **The histogram of the negative pairs' PITA scores and the fitted distribution**. In the training data, 8831 PITA scores for the negative miRNA-target pairs are obtained. Its histogram is fitted with Gaussian distribution. The fitted distribution is represent by blue stars. Click here for file

Additional file 10 **The histogram of the negative pairs' Diana-microT scores and the fitted distribution**. In the training data, 3254 Diana-microT scores for the negative miRNA-target pairs are obtained. Its histogram is fitted with Exponential distribution. The fitted distribution is represent by blue stars. Click here for file
