# Supplementary figures and images for: A Bayesian decision fusion approach for microRNA target prediction
Source: BMC Genomics. 2012 Dec 17;13(Suppl 8):S13. doi: 10.1186/1471-2164-13-S8-S13 (PMC3535698; doi:10.1186/1471-2164-13-S8-S13)

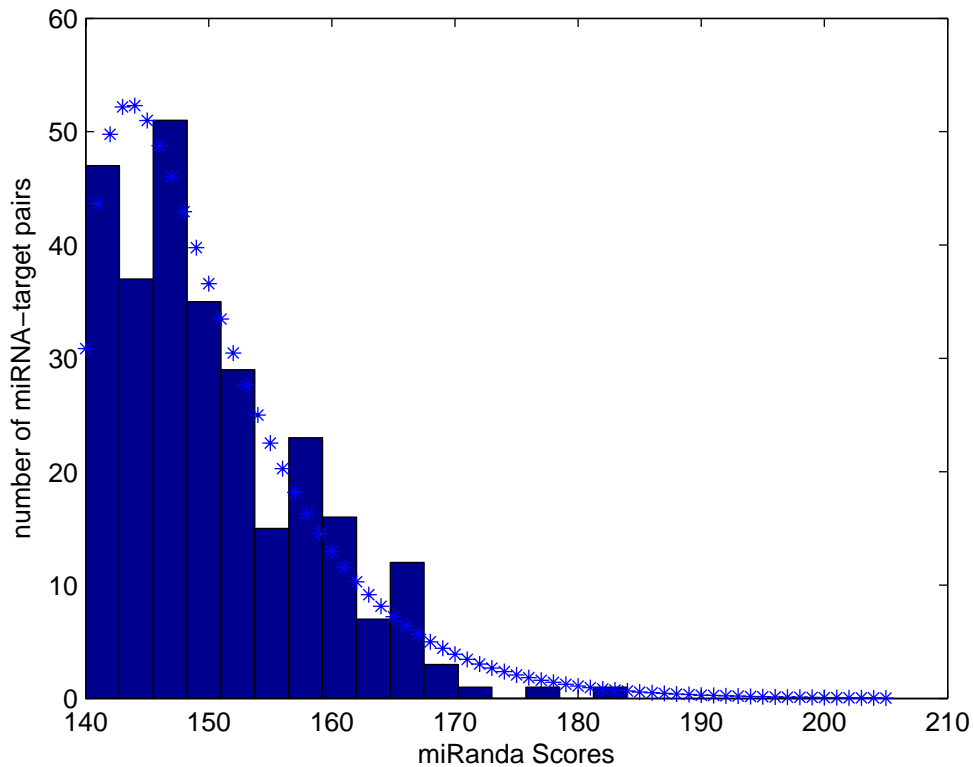

Supplement: Additional file 1 — The histogram of the positive pairs' miRanda scores and the fitted distribution. In the training data, 278 miRanda scores for the positive miRNA-target pairs are obtained. Its histogram is fitted with Negative Binomial distribution. The fitted distribution is represent by blue stars. [file 1471-2164-13-S8-S13-S1.pdf]

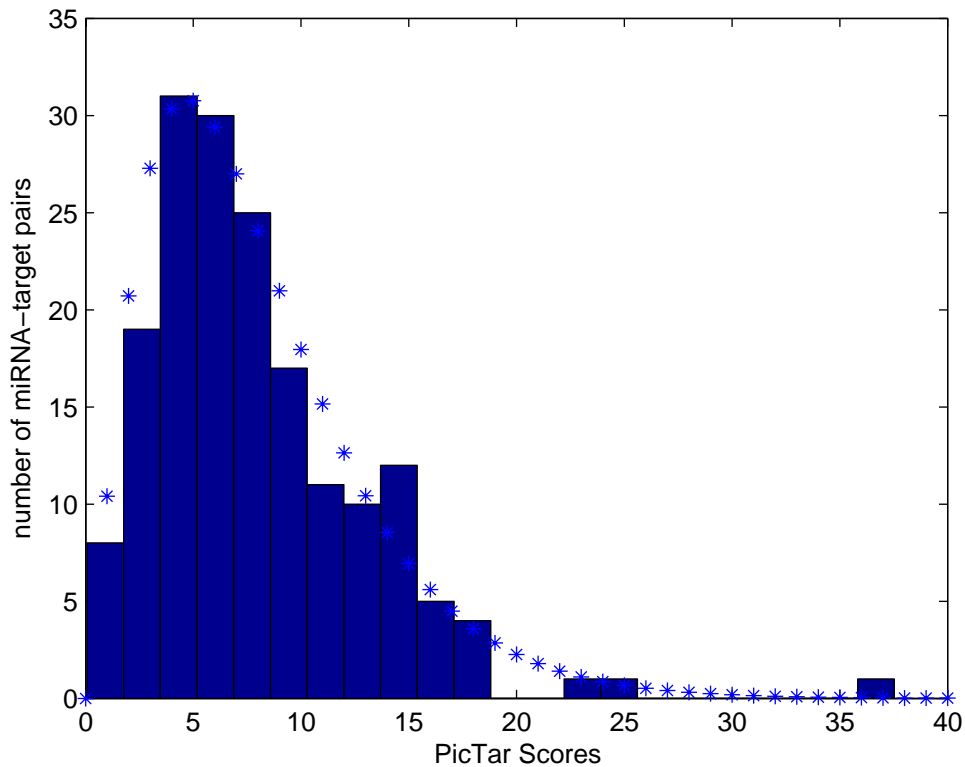

Supplement: Additional file 2 — The histogram of the positive pairs' PicTar scores and the fitted distribution. In the training data, 175 PicTar scores for the positive miRNA-target pairs are obtained. Its histogram is fitted with Gamma distribution. The fitted distribution is represent by blue stars. [file 1471-2164-13-S8-S13-S2.pdf]

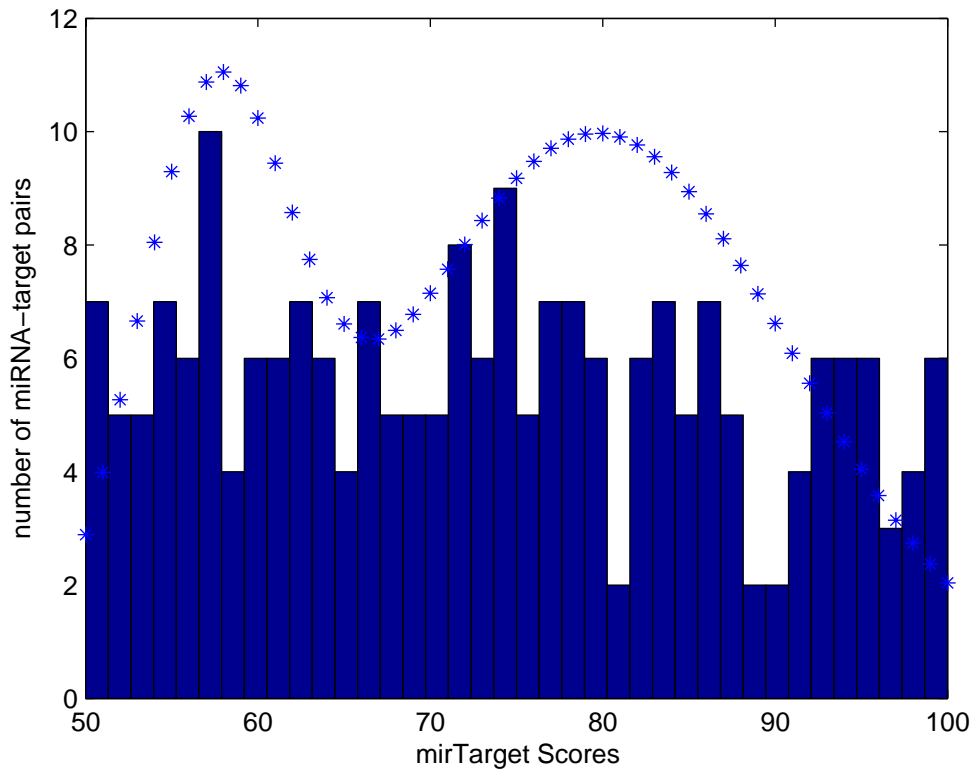

Supplement: Additional file 3 — The histogram of the positive pairs' mirTarget scores and the fitted distribution. In the training data, 214 mirTarget scores for the positive miRNA-target pairs are obtained. Its histogram is fitted with Mixture Gaussian distribution. The fitted distribution is represent by blue stars. [file 1471-2164-13-S8-S13-S3.pdf]

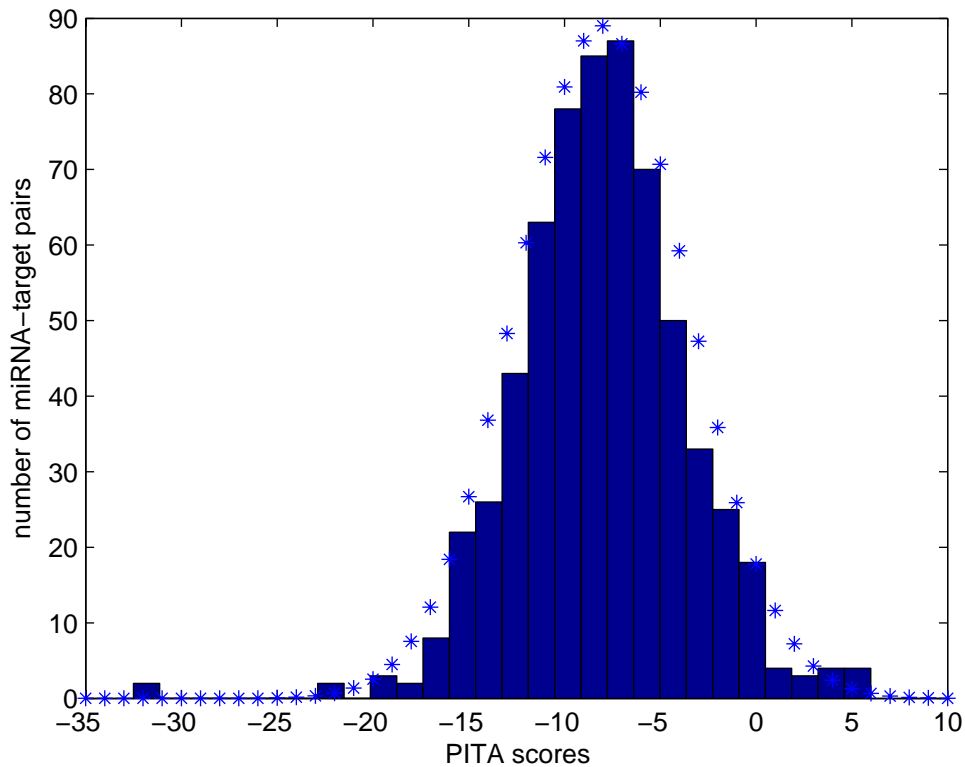

Supplement: Additional file 4 — The histogram of the positive pairs' PITA scores and the fitted distribution. In the training data, 631 PITA scores for the positive miRNA-target pairs are obtained. Its histogram is fitted with Gaussian distribution. The fitted distribution is represent by blue stars. [file 1471-2164-13-S8-S13-S4.pdf]

# Positive

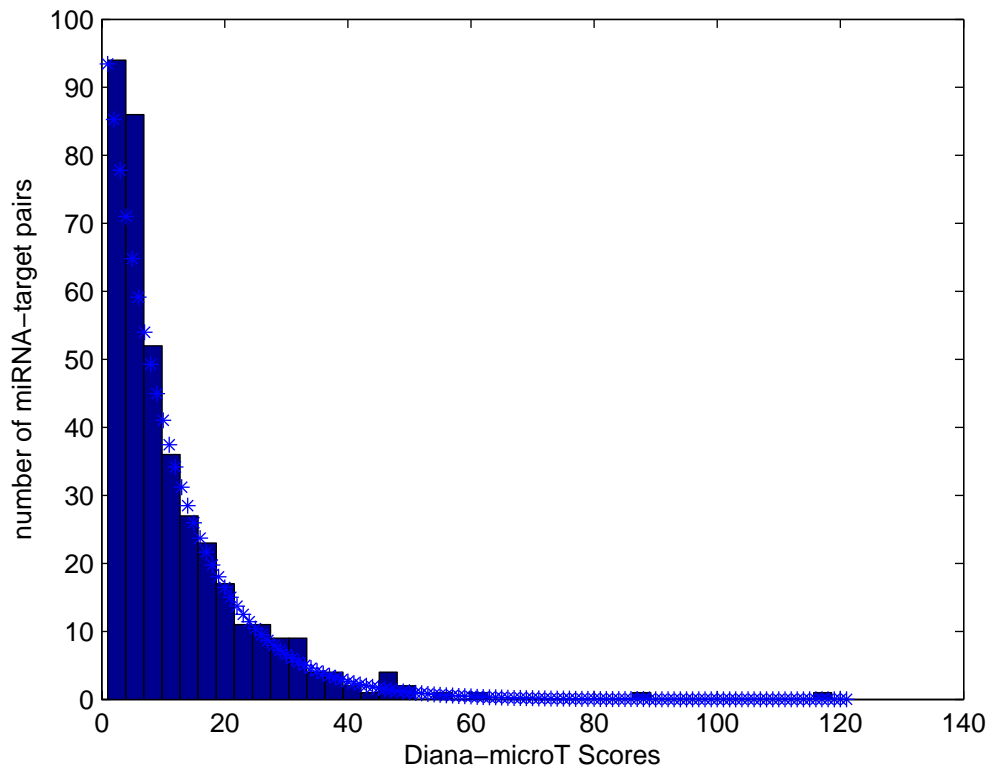

Supplement: Additional file 5 — The histogram of the positive pairs' Diana-microT scores and the fitted distribution. In the training data, 396 Diana-microT scores for the positive miRNA-target pairs are obtained. Its histogram is fitted with Exponential distribution. The fitted distribution is represent by blue stars. [file 1471-2164-13-S8-S13-S5.pdf]

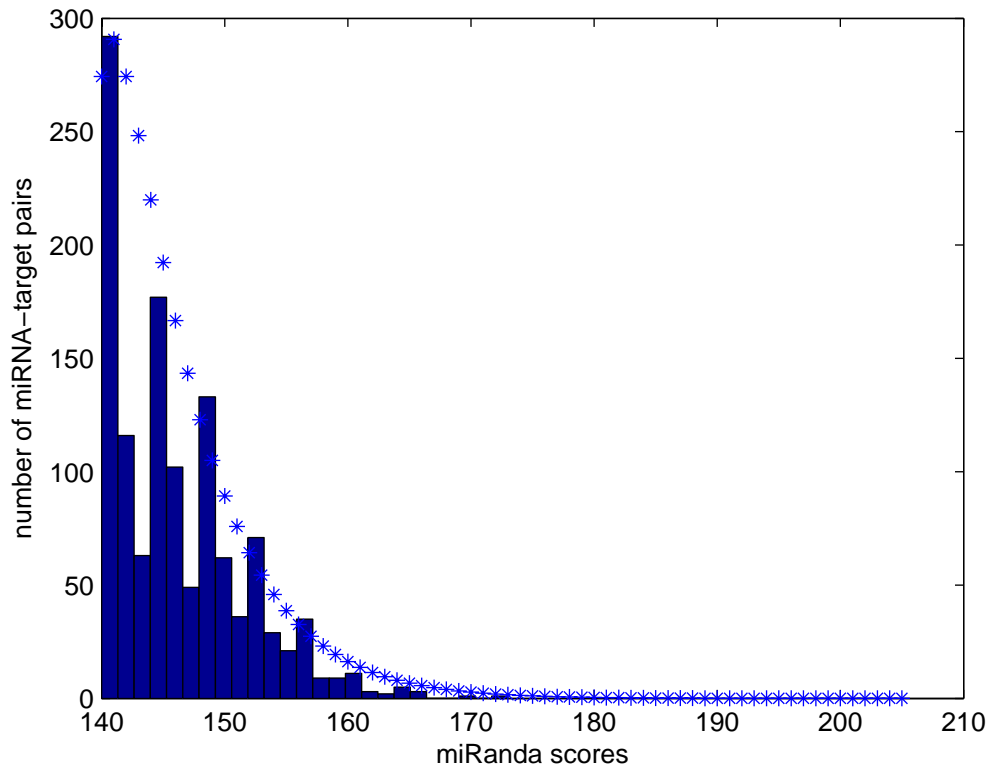

Supplement: Additional file 6 — The histogram of the negative pairs' miRanda scores and the fitted distribution. In the training data, 1230 miRanda scores for the negative miRNA-target pairs are obtained. Its histogram is fitted with Negative Binomial distribution. The fitted distribution is represent by blue stars. [file 1471-2164-13-S8-S13-S6.pdf]

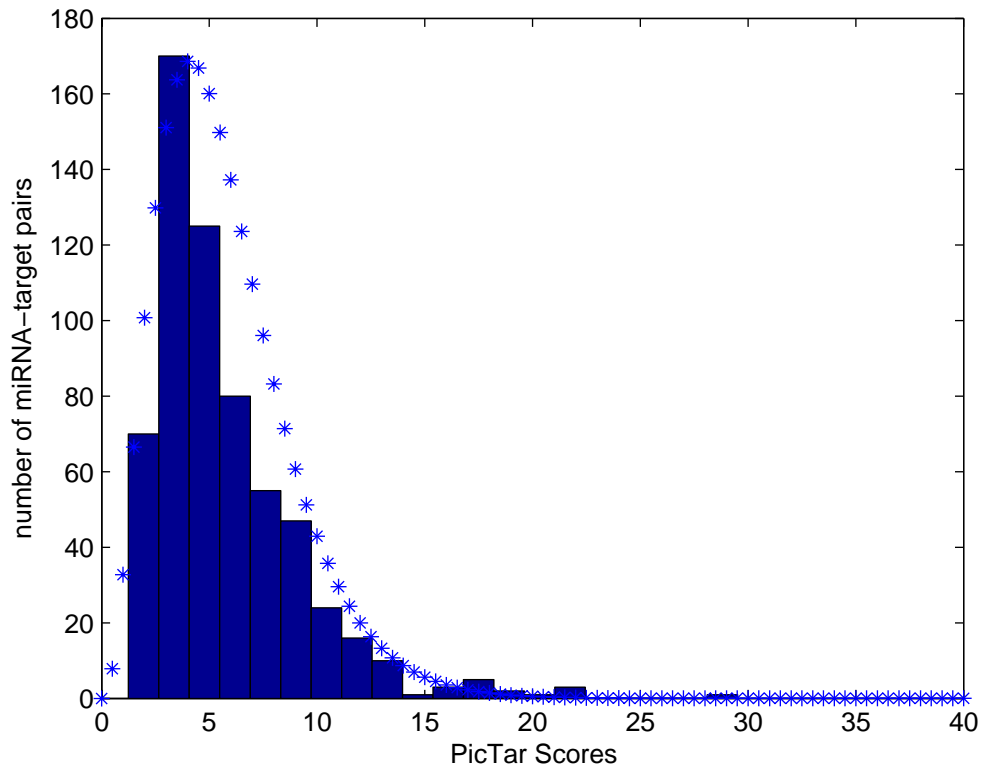

Supplement: Additional file 7 — The histogram of the negative pairs' PicTar scores and the fitted distribution. In the training data, 613 PicTar scores for the negative miRNA-target pairs are obtained. Its histogram is fitted with Gamma distribution. The fitted distribution is represent by blue stars. [file 1471-2164-13-S8-S13-S7.pdf]

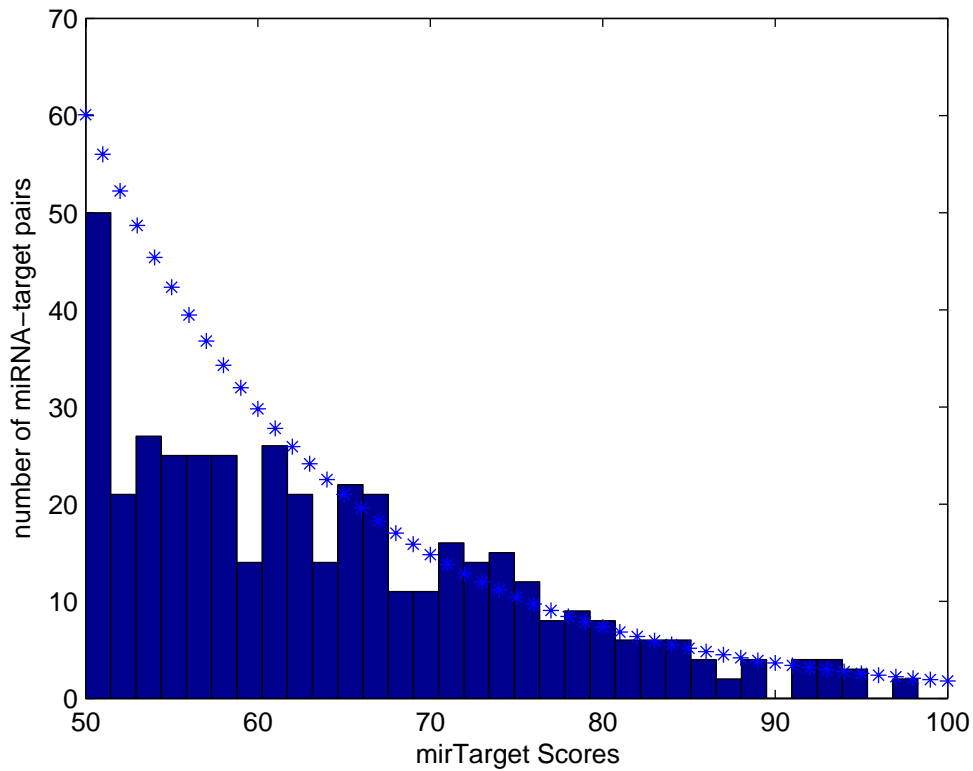

Supplement: Additional file 8 — The histogram of the negative pairs' mirTarget scores and the fitted distribution. In the training data, 436 mirTarget scores for the negative miRNA-target pairs are obtained. Its histogram is fitted with Exponential distribution. The fitted distribution is represent by blue stars. [file 1471-2164-13-S8-S13-S8.pdf]

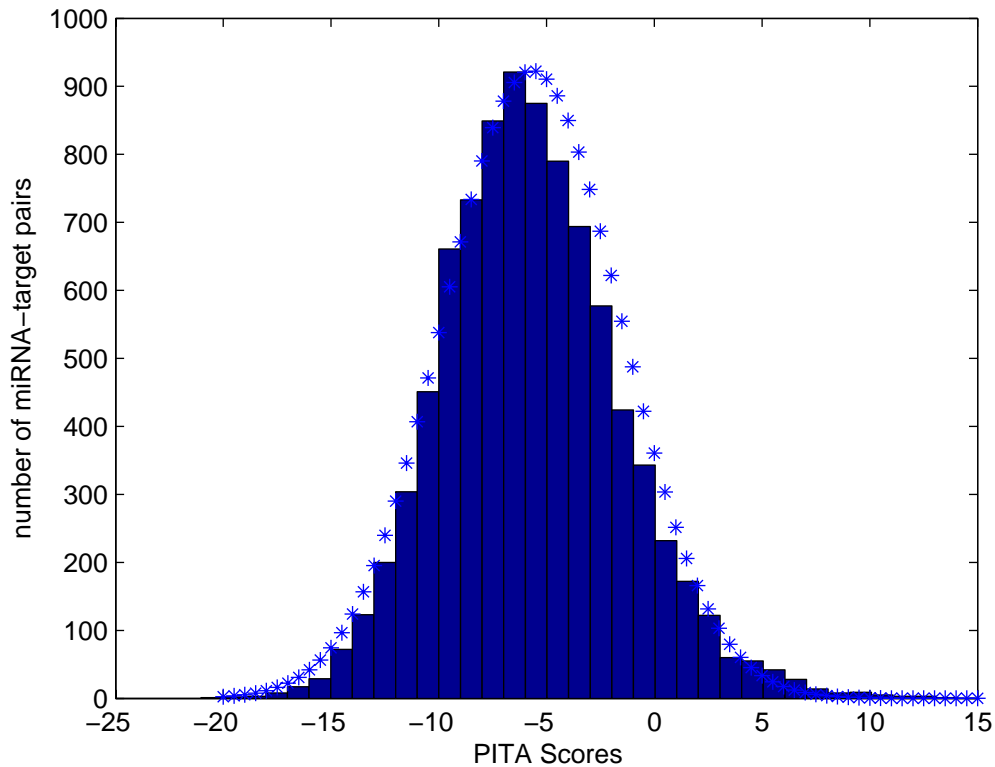

Supplement: Additional file 9 — The histogram of the negative pairs' PITA scores and the fitted distribution. In the training data, 8831 PITA scores for the negative miRNA-target pairs are obtained. Its histogram is fitted with Gaussian distribution. The fitted distribution is represent by blue stars. [file 1471-2164-13-S8-S13-S9.pdf]

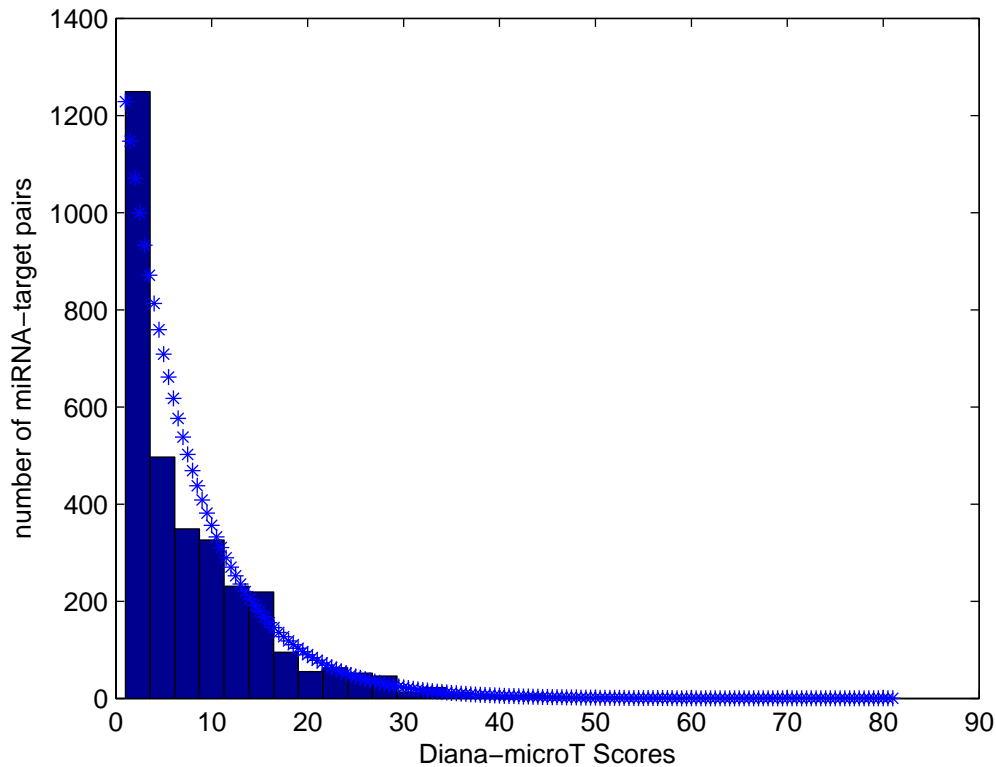

Supplement: Additional file 10 — The histogram of the negative pairs' Diana-microT scores and the fitted distribution. In the training data, 3254 Diana-microT scores for the negative miRNA-target pairs are obtained. Its histogram is fitted with Exponential distribution. The fitted distribution is represent by blue stars. [file 1471-2164-13-S8-S13-S10.pdf]
